# Psychometric properties and validation of the polish version of the Fibromyalgia Impact Questionnaire (FIQ-Pol)

**DOI:** 10.1186/s12889-023-16411-2

**Published:** 2023-08-03

**Authors:** Agnieszka Ćwirlej-Sozańska, Bernard Sozański, Aleksandra Łyko, Anna Łagowska, Natalia Leszczyńska, Barbara Kuduk, Anna Wilmowska-Pietruszyńska

**Affiliations:** 1 Institute of Health Sciences, College of Medical Sciences of the University of Rzeszow, University of Rzeszow, Rejtana 16C, 35-959 Rzeszow, Poland; 2 Institute of Medical Sciences, College of Medical Sciences of the University of Rzeszow, University of Rzeszow, Rejtana 16C, 35-959 Rzeszow, Poland; 3 Faculty of Medicine, Lazarski University, Świeradowska Street 43, 02-662 Warsaw, Poland

**Keywords:** Fibromiyalgia Impact Questionnaire, FIQ-Pol, Fibromyalgia, Disability, Psychometrics

## Abstract

**Background:**

Fibromyalgia (FM) is a chronic condition characterized by widespread musculoskeletal pain, fatigue, intestinal disorders, mood swings, and sleep disturbances. To the best of our knowledge, the questionnaire used for assessing problems and difficulties in the functioning of people with FM has not been translated and adapted in Poland so far. The aim of the study was to assess the psychometric properties of the Polish version of the Fibromyalgia Impact Questionnaire (FIQ-Pol).

**Material and method:**

The study covered 150 people with FM living in Poland. The measurement reliability, internal structure, repeatability, and validity of the Polish version of the FIQ were examined.

**Results:**

The scale score reliability of the entire tool for the research group was very good. The alpha Cronbach's test result for the whole scale was 0.84. The repeatability of the scale measured by the test–retest method using the interclass correlation coefficients (ICC) was very good and amounted to 0.96. Internal structure suggested by FIQ-Pol authors was confirmed (Confirmatory factor analysis). After introducing modification indices for the entire scale, satisfactory parameter values were obtained, i.e.: RMSEA (0.06), CFI (0.97) and TLI (0.96). Theoretical validity was assessed by correlating the results of the Polish version of the FIQ with the results of the Beck's Depression Inventory (BDI). Both the FIQ-Pol total score and its domains showed strong positive correlations with BDI.

**Conclusion:**

The Polish FIQ is a reliable and valid tool to measure the functional disability and health status of Polish people with FM.

## Introduction

Fibromyalgia (FM) is a chronic syndrome characterized by widespread musculoskeletal pain, fatigue, intestinal disorders, mood swings, and sleep disturbances [1]. With reference to the etiology of FM, the phenomenon of central sensitization is emphasized, characterized by impairment of peripheral nerves which are to receive, transmit and process afferent nociceptive stimuli with the predominant manifestation of pain at the level of the musculoskeletal system [2, 3]. In recent years, the pathogenesis of FM has also been associated with inflammatory, immunological, hormonal, genetic and psychosocial factors. Fibromyalgia more often affects women and people aged 30 to 35 [3]. Due to chronic pain, as well as muscle stiffness, depression and anxiety disorders, patients with FM are a special group of patients requiring interdisciplinary therapy.

Despite some progress in understanding the aforementioned pathological process, studies indicate that as many as 75% of patients with FM remain undiagnosed [1]. Due to the lack of specific immunological markers for this disease, the diagnosis of FM is based on clinical assessment in accordance with the American College of Rheumatology criteria (ACR), concerning mainly the subjective feeling of the extent, intensity and duration of pain [4–6].

Very important in the treatment and therapy of patients with FM is the assessment of their limitations and difficulties in everyday functioning. FM negatively affects most aspects of the patient's life, causing extensive functional disability, difficulties in performing daily and work-related activities [7]. The Fibromyalgia Impact Questionnaire (FIQ) is used to assess the functioning difficulties of patients with FM [8–10].

The FIQ was developed to assess the entire spectrum of functional problems faced by patients with FM. The FIQ is widely used in clinical practice and research [10–12]. The instrument is based on three main areas i.e. function, overall impact and symptoms, and is designed to measure those elements of health and functioning that are believed to be most affected by FM. As research shows, FIQ has excellent psychometric properties and allows to distinguish patients with FM from patients with Rheumatoid arthritis (RA), systemic lupus erythematosus or depressive disorders [10–14].

To the best of our knowledge, the questionnaire used for assessing problems and difficulties in the functioning of people with FM has not been translated and adapted in Poland so far. Therefore, there is no thorough assessment of the difficulties in the functioning of people with FM. What's more, there is a lack of epidemiological and clinical studies in Poland indicating key problems in the daily functioning of people with FM.

Therefore, the aim of the study was to assess the psychometric properties of the Polish version of the FIQ.

## Materials and methods

### Study design and participations

This is a cross-sectional study that included people with FM living in Poland. The study was carried out from May to October 2022, and it was performed in cooperation with the College of Medical Sciences of the University of Rzeszow and FIBRO-MY, the National Association of Patients with Fibromyalgia. People diagnosed with FM associated with FIBRO-MY were invited to participate in the study. In order to assess psychometric properties, 150 completed questionnaires were analysed. There were no lost data in the study sample. The necessary sample size was determined based on recommendations [15].

### Procedures

The study was conducted using an online survey distributed among patients associated in FIBRO-MY, the National Association of Patients with Fibromyalgia. Before participating in the study, each person was informed about its objectives and gave informed consent to participate in it.

The following inclusion criteria were adopted: clinical diagnosis of fibromyalgia according to the criteria of the American College of Rheumatology (ACR), age 18 or more, and an informed consent to participate in the study.

The exclusion criteria were cognitive deficits, pregnancy, severe neurological disorders of the central nervous system (e.g., stroke, traumatic brain injury), active cancer, amputations, recent orthopedic injuries, other diseases diagnosed by a physician in the active phase of the course and treatment (e.g., inflammatory rheumatic diseases, acute sciatica) and lack of patient consent.

### Ethics

In accordance with the Declaration of Helsinki, the participants of the study were informed about the purpose and course of the study and gave their informed consent to participate in it. In addition, the consent of the Bioethics Committee of the University of Rzeszow for this study was also obtained (Resolution No. 2022/041).

### Outcome measures

The tool applied in the research was the Fibromyalgia Impact Questionnaire (FIQ). In addition, a study was also conducted among the subjects using the Beck Depression Inventory (BDI). Moreover, the following metrical data were collected: age, gender, place of residence, education, marital status, professional status, type of work performed.

#### The Fibromyalgia Impact Questionnaire (FIQ)

Regarding the translation, cultural adaptation and validation of the FIQ-Pol and its use in scientific research, a license agreement was signed with the Mapi Research Trust dated January 25, 2022 and received order number 2117057.

The first stage was the translation and cultural adaptation of the FIQ-Pol questionnaire in accordance with the Linguistic Validation Guidance of a Clinical Outcome Assessment Mapi Research Trust according to standard recognized methodology of translation [16].

This process included the following steps, i.e., forward translation, backward translation, review by clinicians, cognitive interviews, proofreading. The report on the translation and validation of the Polish version of the FIQ has been approved by the Mapi Research Trust.

The FIQ questionnaire allows us to assess the impact of fibromyalgia on the quality of life of patients. FIQ consists of 20 items examining 10 subscales (scoring dimensions): Physical functioning, Well-being, Work related, Do work, Pain, Fatigue, Rested, Stiffness, Anxiety, Depression. Individual domains are scored according to the conversion key. Once the initial scoring is complete, the scores obtained are converted to normalized scores from 0 to 10, with 0 meaning no impairment and 10 meaning maximum impairment. The total score is expressed on a scale of 0-100. Higher scores mean a greater (negative) impact of the disease on the patient's quality of life. The authors of the original FIQ version confirmed the good psychometric properties of the tool [17].

#### The Back's Depression Inventory (BDI)

The BDI contains 21 items on a 4-point scale ranging from 0 (no symptoms) to 3 (severe symptoms). The reminder period for BDI is 2 weeks. Anxiety symptoms are not assessed, but affective, cognitive, somatic, and vegetative symptoms are considered, reflecting the Diagnostic and Statistical Manual (DSM-IV) criteria for major depression. The minimum score is 0 and the maximum score is 63. Higher scores indicate greater severity of symptoms. In non-clinical populations, scores above 20 indicate depression [18]. In people diagnosed with depression, scores of 0–13 indicate minimal depression, 14–19 (mild depression), 20–28 (moderate depression) and 29–63 (severe depression) [19, 20]. A review of the psychometric properties of the BDI confirmed its good psychometric properties as a self-report measure of depression across settings and populations [21].

### Statistical analysis

Qualitative variables were analysed by calculating the number and percentage of occurrences of each value. The analysis of quantitative variables was performed by calculating the mean, standard deviation, median and quartiles. In order to compare groups, the following tests have been used: with retest and without retest, the chi-square test or Fisher's test were used for qualitative variables, and the Student's t-test (in the case of normal distribution) or the Mann-Whitney test (in the case of lack of normal distribution) for quantitative variables. Normal distribution of quantitative variables was verified by the use of the Shapiro-Wilk test. The analysis adopted a significance level of 0.05.

The analysis was performed in the R program, version 4.2.2. [22].

#### Analysis of the reliability

The analysis was carried out at the level of overall results and at the level of individual domains. The internal consistency of the scale was assessed using Cronbach's alpha coefficient. According to the Nunnally’s criterion, the scale was considered internally consistent if the measure was not <0.7 [23]. The percentage of people with extreme values for each domain was also assessed. The stability of the scale (repeatability) was assessed by the use of the test-retest method, in parallel with the correlation analysis using Spearman's rank correlation coefficient. In addition, the reliability of the test-retest method was assessed using the Intraclass Correlation Coefficient (ICC) [24]. Moreover, Standard Error of Measurement (SEM) and Minimum Detectable Change (MDC) measures were used to determine the amount of measurement error. The study was conducted in a group of 30 people for whom two measurements of the FIQ scale were available. The average interval between two measurements, carried out by two different people, was 7 days.

Confirmatory factor analysis (CFA) was used to check internal structure suggested by FIQ authors. For the Physical functioning subscale, where items 1-11 are expressed on an ordinal scale, the Diagonally Weighted Least Squares estimator was used. For the Total FIQ score, additional modifications indicated by the so-called modification indices were applied. The subsequent measures of fit were used in the analysis: Root Mean Square Error of Approximation (RMSEA), Comparative Fit Index (CFI), Tucker-Lewis Index (TLI) and Standardized Root Mean Residual (SRMR). Following Hu and Bentler, it was assumed that the model is well matched according to Hu and Bentler when we have RMSEA<0.06, CFI and TLI>0.95 and SRMR<0.08. Due to the fact that meeting the conditions for all 4 measures is often too restrictive, the Two-Index Strategy was additionally applied for the assessment, which assumes that the model is well fitted when SRMR<0.09 and additionally one of the conditions CFI>0.96, TLI>0.96 or RMSEA<0.06 takes place [25].

#### Floor and ceiling effects

Floor and ceiling effects were calculated by determining the percentage of participants who obtained the lowest or highest possible scores for each FIQ domain.

#### Analysis of the validity

Convergent validity was assessed by correlating the results of the FIQ questionnaire and the BDI questionnaire. The analysis was performed by examining the Pearson correlation coefficient (when both distributions were normal) or Spearman's test (when at least one distribution was not normal). It has been confirmed that the stronger the depressive symptoms, the greater the impact of fibromyalgia on the quality of life [26–28].

## Results

### Characteristics of the study group

The study covered 150 people aged 18 to 76, including 121 women and 29 men. The average age of the subjects was 44.78 years (SD = 10.77 years). The vast majority of the respondents lived in rural areas (80.67%), and 70% of the respondents declared that they were married or in a partnership. More than half of the respondents (55.33%) had higher education, and more than every third person (33.00%) had secondary education. 57.33% of the participants were professionally active. There were no statistically significant differences between sociodemographic variables and FIQ-Pol scores in the single and double (test-retest) groups (Table 1).

**Table 1:** General socio-demographic characteristics of the study population (*N* = 150)

Parameter	Group with retest (*n*= 30)	Group without retest (*n* = 120)	Total (*N* = 150)	*p*
Sex				
Female	23 (76.67%)	98 (81.67%)	121 (80.67%)	0.717^a)^
Male	7 (23.33%)	22 (18.33%)	29 (19.33%)	
Residence				
City	28 (93.33%)	93 (77.50%)	121 (80.67%)	0.088^b)^
Village	2 (6.67%)	27 (22.50%)	29 (19.33%)	
Age				
Mean (SD)	45.93 (12.22)	44.49 (10.42)	44.78 (10.77)	0.514^c)^
Median (quartiles)	47.00 (35.00–52.00)	45.00 (37.00–52.00)	45.00 (37.00–52.00)	
Range	18.00–45.00	25.00–76.00	18.00–76.00	
Marital status				
Single	3 (10.00%)	14 (11.67%)	17 (11.33%)	0.995^b)^
Married	18 (60.00%)	65 (54.17%)	83 (55.33%)	
Divorced	4 (13.33%)	20 (16.67%)	24 (16.00%)	
Widow/widower	1 (3.33%)	3 (2.50%)	4 (2.67%)	
In a partnership	4 (13.33%)	18 (15.00%)	22 (14.67%)	
Education				
Primary or lower	1 (3.33%)	2 (1.67%)	3 (2.00%)	0.485^b)^
Vocational	2 (6.67%)	8 (6.67%)	10 (6.67%)	
Secondary	8 (26.67%)	46 (38.33%)	54 (36.00%)	
Higher	19 (63.33%)	64 (53.33%)	83 (55.33%)	
Professional status				
Full time employment on open-ended contract	9 (30.00%)	43 (35.83%)	52 (34.67%)	0.662^b)^
Full time employment on fixed-term contract	3 (10.00%)	9 (7.50%)	12 (8.00%)	
Employed on the basis of mandatory contracts or contracts for specific work	1 (3.33%)	7 (5.83%)	8 (5.33%)	
Self-employment	4 (13.33%)	10 (8.33%)	14 (9.33%)	
Annuitant	3 (10.00%)	12 (10.00%)	15 (10.00%)	
Pensioner	3 (10.00%)	4 (3.33%)	7 (4.67%)	
Unemployed	7 (23.33%)	35 (29.17%)	42 (28.00%)	
Total FIQ-Pol score				
Mean (SD)	73.12 (15.85)	74.24 (13.24)	74.02 (13.75)	0.905^d)^
Median (quartiles)	77 (67.83–85.07)	76.02 (65.46–83.51)	76.08 (65.92–84.43)	
Range	26.12–95.48	30.77–99.09	26.12–99.09	
FIQ-Pol: Physical functioning				
Mean (SD)	5.3 (2.13)	5.22 (1.89)	5.24 (1.94)	0.772^d)^
Median (quartiles)	5.51 (3.94–6.67)	5.45 (4.24–6.67)	5.45 (4.24–6.67)	
Range	0–9.7	0–9.09	0–9.7	
FIQ-Pol: Well-being				
Mean (SD)	8.14 (2.79)	7.86 (2.29)	7.91 (2.39)	0.241^d)^
Median (quartiles)	10 (7.14–10)	8.57 (7.14–10)	8.57 (7.14–10)	
Range	0–10	0–10	0–10	
FIQ-Pol: Work related				
Mean (SD)	4.81 (3.34)	5.46 (3.05)	5.33 (3.11)	0.332^d)^
Median (quartiles)	4.29 (1.79–7.14)	5.71 (2.86–7.14)	5.71 (2.86–7.14)	
Range	0–10	0–10	0–10	
FIQ:-Pol Difficulties at work				
Mean (SD)	7.97 (2.03)	7.71 (2.01)	7.76 (2.01)	0.406^d)^
Median (quartiles)	8 (8–9)	8 (7–9)	8 (7–9)	
Range	1–10	2–10	1–10	
FIQ-Pol: Pain				
Mean (SD)	7.93 (1.93)	7.65 (1.76)	7.71 (1.79)	0.301^d)^
Median (quartiles)	8 (7–9)	8 (7–9)	8 (7–9)	
Range	2–10	2–10	2–10	
FIQ-Pol: Fatigue				
Mean (SD)	8.8 (1.49)	8.88 (1.22)	8.86 (1.27)	0.843^d)^
Median (quartiles)	9 (8–10)	9 (8–10)	9 (8–10)	
Range	5–10	5–10	5–10	
FIQ: Rested				
Mean (SD)	8.53 (2.05)	8.69 (1.93)	8.66 (1.94)	0.606^d)^
Median (quartiles)	9 (8–10)	9 (8–10)	9 (8–10)	
Range	2–10	1–10	1–10	
FIQ-Pol: Stiffness				
Mean (SD)	8.27 (1.87)	8.33 (1.82)	8.32 (1.82)	0.917^d)^
Median (quartiles)	9 (7–10)	9 (7–10)	9 (7–10)	
Range	4–10	3–10	3–10	
FIQ-Pol: Anxiety				
Mean (SD)	6.83 (2.88)	7.47 (2.03)	7.34 (2.23)	0.525^d)^
Median (quartiles)	8 (5–9)	8 (6–9)	8 (6–9)	
Range	1–10	1–10	1–10	
FIQ-Pol: Depression				
Mean (SD)	6.53 (2.97)	6.97 (2.46)	6.89 (2.57)	0.617^d)^
Median (quartiles)	7.5 (4–8.75)	7 (5–9)	7 (5–9)	
Range	1–10	1–10	1–10	
BDI				
Mean (SD)	25.87 (11.56)	26.55 (10.85)	26.41 (10.96)	0.778^d)^
Median (quartiles)	26.5 (19–32.75)	26 (19–34)	26 (19–33.75)	
Range	5–54	0–50	0–54	

^a)^Chi-square test

^b)^Fisher's exact test

^c)^Student's t-test for independent samples

^d)^Mann–Whitney test

### Reliability analysis

#### Internal consistency - Cronbach's alpha

Since the FIQ-Pol has a "2-step" computational structure, items 1-11 were first combined into a single Physical functioning construct, and then this construct, together with the remaining questions, form the FIQ-Pol total score. Therefore, Cronbach's alpha and CFA were calculated first for the Physical functioning subscale (in order to test whether items 1-11 really make a valid construct) and then the same was performed for the total score (where the Physical functioning subscale was shown as a "single item").

##### Physical functioning subscale

Cronbach's alpha for the Physical functioning subscale amounted to 0.928, which means that the subscale is reliable.

All items in the Physical functioning subscale have positive discriminant power. This means that they correlate positively with the other items included in the scale, which is a very desirable effect. Excluding any of the items does not increase Cronbach's alpha coefficient, which means that the scale is well constructed (Table 2).

**Table 2:** Analysis of internal consistency for the Physical functioning subscale

Item	Cronbach's alpha after item exclusion	Discriminant power
1	0.923	0.664
2	0.919	0.744
3	0.919	0.768
4	0.921	0.719
5	0.918	0.748
6	0.921	0.713
7	0.919	0.679
8	0.920	0.728
9	0.921	0.683
10	0.914	0.700
11	0.923	0.679

##### FIQ-Pol total score

Cronbach's alpha for the entire scale is 0.837, which means that the FIQ-Pol is reliable, and its results are repeatable. All items of the scale have positive discriminant power. Excluding any of the items does not increase Cronbach's alpha coefficient, which means that the scale is well constructed (Table 3).

**Table 3:** Internal consistency analysis for FIQ-Pol total score

Item	Cronbach's alpha after item exclusion	Discriminant power
1	0.822	0.531
2	0.830	0.453
3	0.833	0.493
4	0.804	0.725
5	0.813	0.652
6	0.821	0.623
7	0.835	0.381
8	0.825	0.495
9	0.814	0.608
10	0.821	0.550

#### Test-retest analysis and measurement errors

Interclass correlation coefficients (ICC) are very high (about 0.9 or higher) for all subscales except well-being and fatigue, where it is only slightly lower. This means that FIQ-Pol scores are stable. The measures of measurement error, SEM and MDC, are the highest for the total score and the lowest for anxiety and depression subscales (Table 4).

**Table 4:** Test–retest analysis

FIQ-Pol	ICC (95%CI)	SEM	MDC
FIQ-Pol Total Score	0.955 (0.906—0.978)	3.20	8.87
Physical functioning	0.945 (0.888—0.973)	0.47	1.30
Well-being	0.845 (0.699—0.924)	0.99	2.74
Work related	0.894 (0.791—0.948)	1.03	2.86
Do work	0.936 (0.858—0.970)	0.53	1.47
Pain	0.902 (0.803—0.953)	0.61	1.69
Fatigue	0.800 (0.622—0.899)	0.66	1.83
Rested	0.926 (0.852—0.964)	0.56	1.55
Stiffness	0.955 (0.907—0.979)	0.39	1.08
Anxiety	0.986 (0.972—0.993)	0.34	0.94
Depression	0.987 (0.973—0.994)	0.34	0.94

#### Internal structure

Due to the fact that the correlations between the subscales and the FIQ-Pol total score are not a simple sum of domains, CFA was used to assess the internal structure.

##### Physical functioning subscale

With reference to the subscale of Physical functioning, very good values of RMSEA (<0.001), CFI and TLI (>0.999), SRMR (0.062) were obtained, which confirm the good structure of this tool (Table 5).

**Table 5:** Confirmatory factor analysis for Physical functioning subscale

Chi-square test			RMSEA	CFI	TLI	SRMR
**χ**^**2**^	**df**	**p**				
20.607	44	0.999	< 0.001	> 0.999	> 0.999	0.062

##### FIQ-Pol total score

Regarding the total score, unsatisfactory fit indices RMSEA, CFI, TLI and SRMR were obtained (model I in Table 6). Since no direct confirmation of the construction of the total score was obtained, some modifications were applied indicated by the so-called modification indices. In this case, they suggest introducing correlations between the following item pairs into the model: item 14 and item 15, item 15 and item 18, item 17 and item 18, item 19 and item 10.

**Table 6:** Confirmatory factor analysis for the total score

Model	chi-square test			RMSEA	CFI	TLI	SRMR
	**χ**^**2**^	**df**	***p***				
I	200.711	35	< 0.001	0.178	0.728	0.65	0.094
II	47.066	31	0.032	0.059	0.974	0.962	0.045

The introduced modification indices allowed for obtaining the desired parameter values (SRMR<0.09, CFI>0.96, TLI>0.96, RMSEA<0.06 (model II in Table 6). Therefore, the construction of the total result was confirmed, with the condition that the answers to the questions listed are strongly related to each other.

### Floor and ceiling effects

Floor and ceiling effects do not exceed 50%. The highest floor effect was found for item 12 (42.0%), and the highest ceiling effect for item 17 (44.7%) (Table 7).

**Table 7:** Floor and ceiling effects

Item	Floor effect	Ceiling effect	Not applicable
1	5.3%	4.7%	2.7%
2	24.7%	2.0%	2.0%
3	10.0%	3.3%	0.0%
4	7.3%	4.7%	6.0%
5	6.0%	14.7%	10.7%
6	14.7%	6.7%	4.0%
7	12.0%	17.3%	8.0%
8	5.3%	28.0%	5.3%
9	6.7%	24.7%	16.7%
10	13.3%	14.0%	28.0%
11	12.7%	10.7%	2.7%
12	42.0%	2.0%	0.0%
13	11.3%	13.3%	0.0%
14	0.0%	21.3%	0.0%
15	0.0%	17.3%	0.0%
16	0.0%	42.0%	0.0%
17	0.0%	44.7%	0.0%
18	0.0%	34.0%	0.0%
19	0.0%	18.0%	0.0%
20	0.0%	18.7%	0.0%

### Theoretical validity

#### Convergent validity

Convergent validity was tested by correlating the obtained FIQ-Pol scores with the results of the BDI questionnaire (Table 8).

**Table 8:** FIQ and BDI correlation analysis

FIQ-Pol	BDI
	**Spearman's correlation coefficient**
FIQ-Pol Total Score	*r* = 0.640. *p* < 0.001*
Physical functioning	*r* = 0.445. *p* < 0.001*
Well-being	*r* = 0.319. *p* < 0.001*
Work related	*r* = 0.396. *p* < 0.001*
Do work	*r* = 0.341. *p* < 0.001*
Pain	*r* = 0.381. *p* < 0.001*
Fatigue	*r* = 0.418. *p* < 0.001*
Rested	*r* = 0.230. *p* = 0.005*
Stiffness	*r* = 0.215. *p* = 0.008*
Anxiety	*r* = 0.572. *p* < 0.001*
Depression	*r* = 0.716. *p* < 0.001*

^*^statistically significant relationship (*p* < 0.05)

BDI correlates significantly (*p*˂0.05) and positively (r˃0) with the FIQ-Pol total score and with each of its subscales, so the higher the BDI score (stronger depressive symptoms), the greater the impact of fibromyalgia on the quality of life in all areas.

## Discussion

Adaptation of measurement tools gives the possibility of global exchange of scientific results in a standardized way and allows for their reliable comparison in the international arena [29–32].

The FIQ is one of the most commonly used instruments capable to measure the current health status of patients with FM, which (covering the entire spectrum of problems of this disease) is highly comprehensive. Until now, it has been translated and approved into several languages, including Swedish [31] Hebrew [32], German [33], Turkish [34], Korean [35], French [36], Italian [37], Spanish [38], Portuguese [39], Arabic [40], Dutch [41] and Finnish [42]. To our knowledge, this study is the first attempt to validate the FIQ into Polish. As the results show, the Polish version of the FIQ questionnaire is a reliable and valid measure of the health status of patients with FM.

After the process of cultural and linguistic adaptation, the psychometric properties of the questionnaire were verified. The sample of patients with FM in the Polish study presents the expected demographic characteristics and is very similar to those used in other studies, such as one of the Spanish validations from 2013 [43] or the Chinese validation from 2023 [44], i.e. the subjects were mainly married middle-aged women with higher education, usually working intellectually or not working due to poor health.

The total FIQ score in the Polish version for the study sample was on average 74.02 (SD = 13.75). These values are slightly higher but still similar to those obtained by Srifi et al. in the Moroccan version (65.00 SD: 14.5) [45] or by Salgueiro et al. in the Spanish version (68.22, SD: 14.5) [43]. The average total score in the Polish version is higher compared to the Brazilian version [46], in which it was (61.2, SD: 24.3) and much higher than the Finnish version [42], in which it was (49.8, SD:19.9).

Considering the results in individual subscales in the Polish sample, it was found that FM had the greatest impact in such areas as: Fatigue (8.86, SD: 1.27), Rested (8.66, SD: 1.94) and Stiffness (8.32, SD: 1,82). These results are most similar to the results of the Spanish study [42], in which the following areas were rated the highest: Fatigue (7.73, SD: 2.3), Rested (8.62, SD: 2,1) and Body stiffness (7.39, SD: 2,2). On the other hand, FM had the least importance in the following areas: Work related (5.33, SD: 3.11), Depression (6.89, SD: 2.57) and Physical functioning (5.24, SD: 1.94), and these results are comparable to the Dutch study [41], where these areas showed the lowest values: Work related (5.7, SD: 2.1), Depression (2.7, SD: 2.4) and Physical functioning (4 .5, SD: 1.6).

The internal consistency of the FIQ-Pol in the subscale of Physical functioning was assessed by the use of the Cronbach's alpha coefficient at the level of 0.93, which indicates high reliability of the scale. Cronbach's alpha coefficient for this subscale in other language versions ranged from 0.86 in the Spanish version [38] to 0.90 in the Chinese version [44] and 0.91 in the Dutch version [41].

With regard to the total FIQ-Pol score, Cronbach's alpha in this scale was 0.84, which indicates good consistency between the questions and proves the high reliability of the scale. This level of internal consistency is slightly lower than the original English version [47] amounting 0.95 or the Portuguese version (0.94) [48], but this is not a significant difference, and the internal consistency of the whole scale was also comparable to previous versions, such as Spanish (0.91) [43], Turkish (0.89) [49], Japanese (0.90) [50] or Bengali (0.83) [51].

Repeatability of the scale was measured by the test-retest method using interclass correlation coefficients. The ICC scores for all subscales were very high. The ICC for the total FIQ-Pol score was 0.96, similar to the Arabic (0.93) [40], Japanese (0.91) [50] and Chinese (0.90) [44] versions. In the Spanish version, this result was lower, but still comparable (0.82) [43]. The ICC for the subscale of Physical functioning was 0.95, while in the study carried out by Isomura et al. the result was 0.84 [50] and in the study performed by Abu-Dahab et al. it was 0.83 [40] respectively.

Confirmatory factor analysis was used to assess internal consistency. In our study, we calculated the CFA based on the structure of the questionnaire presented by the authors of the FIQ. After including the modification indices, the desired parameter values were obtained, i.e. RMSEA (0.06), CFI (0.97) and TLI (0.96). Other authors made a slightly different division of the FIQ structure, probably at their own discretion, but they also confirmed good internal consistency, obtaining similar values of individual indicators. Li et al. got the following results: RMSE (0.08) and CFI (0.90) respectively, but the study does not provide information on the TLI value [44]. The results can also be compared to the study carried out by Luciano et al. where the results reached the values for RMSE (0.06), CFI (0.93) and TLI (0.94) [52]. The results of our own study were also compared to the Revised FIQ version by authors Lupi et al. They obtained comparable results for RMSEA (0.06), CFI (0.93) and TLI (0.93) [53]. The results of CFA were also referred to the work of Salaffi et al. in which the authors also dealt with the Revised FIQ. The results were 0.11 for RMSEA, 0.95 for CFI and 0.94 for TLI, respectively [54].

Few FIQ validation studies have evaluated the effect of the floor and ceiling. In this respect, the Polish version can be compared to the Finnish version [42]. The results for the Polish version, both for the floor and ceiling effects, do not exceed 50%. Such a value means that these effects do not occur, which is good information and indicates the correct selection of questions for the research population. Regarding the Finnish version, floor and ceiling effects ranged on average from 1% to 75% and from 0% to 27%.

Moreover, theoretical validity was also assessed by correlating the results of the Polish version of the FIQ with the results of other instruments that were consistently associated with FM, such as depressive symptomatology assessed by the BDI. As previously observed [44, 48], both the FIQ total score and its domains presented strong positive correlations with the BDI. The above relationship confirms that psychological factors, especially depression, play an important role in health-related quality of life in patients with FM [55].

### Research limitations and further research directions

From the statistical point of view, the size of the group could be considered a limitation of the study. The greater the number, the greater the certainty of the measurement. In addition, in future research, it would be possible to assess the accuracy of the content for the Polish version of the FIQ. Further studies are also needed to determine the sensitivity of FIQ-Pol to changes in the clinical status of patients with fibromyalgia.

## Conclusions

The FIQ-Pol is a reliable and valid tool for measuring the functional disability and health status of Polish patients with FM. Thus, the FIQ-Pol can be used in clinical settings and for research purposes.

## Data Availability

The datasets used and analyzed in the current study are available from the corresponding author on reasonable request.
